# Chemobrain Experienced by Breast Cancer Survivors: A Meta-Ethnography Study Investigating Research and Care Implications

**DOI:** 10.1371/journal.pone.0108002

**Published:** 2014-09-26

**Authors:** Maryam Hafsah Selamat, Siew Yim Loh, Lynette Mackenzie, Janette Vardy

**Affiliations:** 1 Department of Postgraduate Studies, University of Malaya, Kuala Lumpur, Malaysia; 2 Department of Rehabilitation Medicine, Faculty of Medicine, University of Malaya, Kuala Lumpur, Malaysia; 3 Discipline of Occupational Therapy, Faculty of Health Sciences, University of Sydney, Sydney, Australia; 4 Concord Cancer Centre, Concord Repatriation and General Hospital, Concord, Sydney, Australia; 5 Sydney Medical School, The University of Sydney, Sydney, Australia; Northwestern University Feinberg School of Medicine, United States of America

## Abstract

**Background:**

Cognitive impairment, colloquially termed “chemobrain”, occurs in 10–40% of all cancer patients, and is an emerging target of cancer survivorship research.

**Aim:**

This study reviews published qualitative studies to explore cognitive impairments or chemobrain among breast cancer survivors, with particular attention given to the impact on quality of life.

**Method:**

Using keywords, we searched ten electronic databases (CINAHL, EMBASE, Proquest, OVID SP, MEDLINE, Oxford Journal, Science Direct, PubMED).

**Findings:**

Of 457 papers, seven relevant papers were included. Data was extracted and concepts were analysed using a meta ethnography approach. Four second order intepretations were identified, on the basis of which, four third order intrepretations were constructed. Linked together in a line of argument, was a consistent account on their struggles to self-manage the chemobrain impairments that impact their daily lives. Five concepts emerged from the analysis of the primary findings: i) real experiences of cognitive changes, ii) calls for help, iii) impact of cognitive impairments, iv) coping and v) survivorship and meaning. Further synthesis resulted in four new order intepretations: i) The chemobrain struggle, ii) The substantial impact of chemobrain on life domains, iii) The struggle to readjust and to self manage, and iv) ‘thankful yet fearful’ representation.

**Discussion:**

Awareness of cognitive changes were context-dependent on healthcare settings and cultural contexts as strong determinants. Subjects verified the existence of chemobrain but healthcare providers mis-recognised, under-recognised, and sometimes negated it perhaps due to its unknown aetiology. Asian breast cancer survivors appear less vocal than their western counterparts.

**Conclusion:**

The current literature on the lived experiences of how women experienced chemobrain provides a consistent report that chemobrain is real, persistent and with detrimental impacts on quality of life - manifested as a constant struggles. A greater awareness of the effects of chemobrain with improved functional assessment and interventions is warranted.

## Introduction

Cancer survivorship is an emerging field of study and development. Today, there is a steep rise in numbers of cancer survivors internationally, due to earlier detection, better chemo-therapeutic regimens and multidisciplinary collaborative care approaches [1], [2]. In the United State alone, the 5-year relative survival rate for female breast cancer has improved significantly from 63% in the early 1960s to 90% in 2011 [3]. With the rise in survivors, attention is turning to studying the longer-term adverse effects of treatment and the impact they can have on daily activities of living, participation and functioning by occupational therapists. The phenomena of cognitive impairment after cancer, is gaining increasing attention as one of the key foci of cancer survivorship research by health professionals.

Cognitive impairment is being acknowledged as an after-effect of cancer treatment, and is also commonly known as ‘chemofog’ or ‘chemobrain’ [4]–[9]. Matsuda et al. [7] reported that chemotherapy-induced cognitive deficits occurred in 10–40% of all cancer patients, with up to 23% in women with breast cancer [6]. Overall, the estimated prevalance of chemobrain varies in the literature from 15% to 70% [10]. The aetiology of chemobrain remains unknown. However, there are indications that it may be due to toxicity from chemotherapy agents, especially high dose treatments [11]. Hypotheses of the mechanisms involved include vascular injuries and oxidative damage, inflammation, direct injuries to neurons, autoimmune responses, chemotherapy-induced anaemia and the presence of the apolipoprotein Eε4 (APOE ε4) allele [12]. Studies also suggest cognitive changes may be due to a combination of psychological and medical factors associated with adjuvant systemic therapy (e.g. low oestrogen and progesterone from chemotherapy) as well as anticancer hormonal treatments (e.g. tamoxifen or aromatase inhibitors) [10], [13], [14]. Cognitive impairments have also been shown to occur prior to chemotherapy, making it difficult to determine what is actually due to the chemotherapy [6], [14], [15]. Cognitive performance can also be influenced by common problems faced by cancer survivors that may include pain, insomnia, depression and fatigue [16].

Many survivors of breast cancer complain of increased difficulties with multi-tasking and slower mental processing time, which become more noticeable once they try to resume their normal activities. This is especially evident when they return to work and particularly for those in intellectually demanding occupations [17]. In a study by Wagner et al. [18] 63% of cancer survivors reported problems with concentration and attention, 50% problems with memory, and 38% problems with abstract reasoning. The review by Matsuda et al. [7] identified cognitive impairments related to memory loss and inattention [15]; and problems with concentration, visuo-spatial skills, and motor function [6]. This had detrimental consequences on work performance (75%) that required patients to utilize compensatory strategies (58%), with accompanying patient frustration (50%) as well as adverse impacts on family relationships (33%) [18]. Overall these studies highlighted that cancer survivors commonly reported greater difficulties in work related activity with either a decrease in functional ability or maintenance of functional ability levels that required increased mental effort [19].

However, none of these studies captured the lived experiences or the levels of severity of chemobrain symptoms experienced by cancer survivors. It is therefore important for several reasons to undertake an in-depth exploration of this issue, to understand the cognitive changes experienced by breast cancer survivors. Firstly: i) due to high survival rates, breast cancer patients are likely to live with these problems for a considerable time – making it highly pertinent to explore the impact of these cognitive impairments on their quality of life. Secondly, there is no published meta-review of qualitative studies addressing the chemobrain experience. A qualitative review is a relevant approach to evaluate the meaning that breast cancer survivors ascribe to cognitive changes, and it represents an area of concern that would not be feasible to be examined using quantitative approaches. Qualitative approach variables are flexible and better able to explore specific relationships while taking into account the complexity of individual contexts [20]–[22]. Therefore, the aim of this study was to review qualitative studies that explored the lived experience of chemobrain among breast cancer survivors, with particular attention given to the impact of chemobrain on daily living and quality of life.

## Methods

This study is the first part of a larger study involving focus group on survivors. The ethic to conduct the study was approved by University Malaya Ethical Committee Boards. Using a meta-ethnography method, the interpretation of results from a range of original studies were compared and translated to acquire a greater understanding of the cognitive changes experienced by breast cancer survivors [22]–[25]. We used the 7–step process of meta-ethnography by Noblit and Hare [25] -: 1. Developing a specific research question; 2. Deciding what is relevant to the research question; 3. Reviewing each study to identify key concepts and recording them; 4. Determining how the studies are related, and comparing study approaches to the key concepts; 5. Translating the studies into one another; 6. Synthesising the translation outcomes, and 7. Expressing the synthesis.

Step 5 above involved the researchers interpreting and translating the key concepts via:- i) reciprocal translation (i.e. studies with similar findings were directly compared and synthesised), ii) refutational translation (i.e. studies rebutting each other with conflicts of findings), and iii) Lines of Argument (i.e. translation of the studies to build a final interpretation using both differences and similarities among studies) [23].

Based on these steps, the researchers (MHS &SYL) independently appraised the selected papers and undertook the steps to extract the information and synthesize the findings. Further discussions took place between the reviewers when any discrepancies arose. Next, second order intepretations were identified, on the basis of which, third order intrepretations (based on key concepts and second-order interpretations) were constructed. These were all linked together in a line of argument [(i.e. the final interpretation using both differences and similarities among studies) to present the intrepretation of cognitive impairments experienced by women. The synthesis can be expressed as text as well as in summary tables, diagrams or models, which can produce significant new insights into the topic [23]. Finally a table synthesis of concepts and order interpretation was built to connect each of the papers.

### Search strategy

We undertook a systematic search of the literature for all published English language articles from 2002–2013 that used qualitative methods to investigate post-chemotherapy cognitive impairments for women with breast cancer. The following databases were searched: CINAHL, Web of Knowledge, EMBASE, Proquest, OVID SP, MEDLINE, Oxford Journal, Science Direct, PubMED, and Wiley.

Researchers performed free text searches by using keywords of chemobrain OR (chemotherapy AND mild cognitive impairment) OR post-chemotherapy cognitive changes. Next, we add in, the term “ breast cancer survivors” AND qualitative. The search filters were used at this stage to address the inclusion criteria for the study.

The inclusion criteria were: 1) breast cancer and chemobrain. The terms used were chemofog, cognitive dysfunction, cognitive changes. Chemobrain was referred to as any domain of cognition decline or dysfunction experienced by survivors who had chemotherapy; 2) qualitative study. Terms used included lived experience and qualitative experience; 3) studies published from 2002 to 2014. All papers published within the last 10 years; 4) English language text and; 5) full text publication. The exclusion criteria included: 1) study design other than a qualitative design methodology; 2) studies with patients with cancers other than breast cancer; and 3) non English papers.

**Figure 1:**
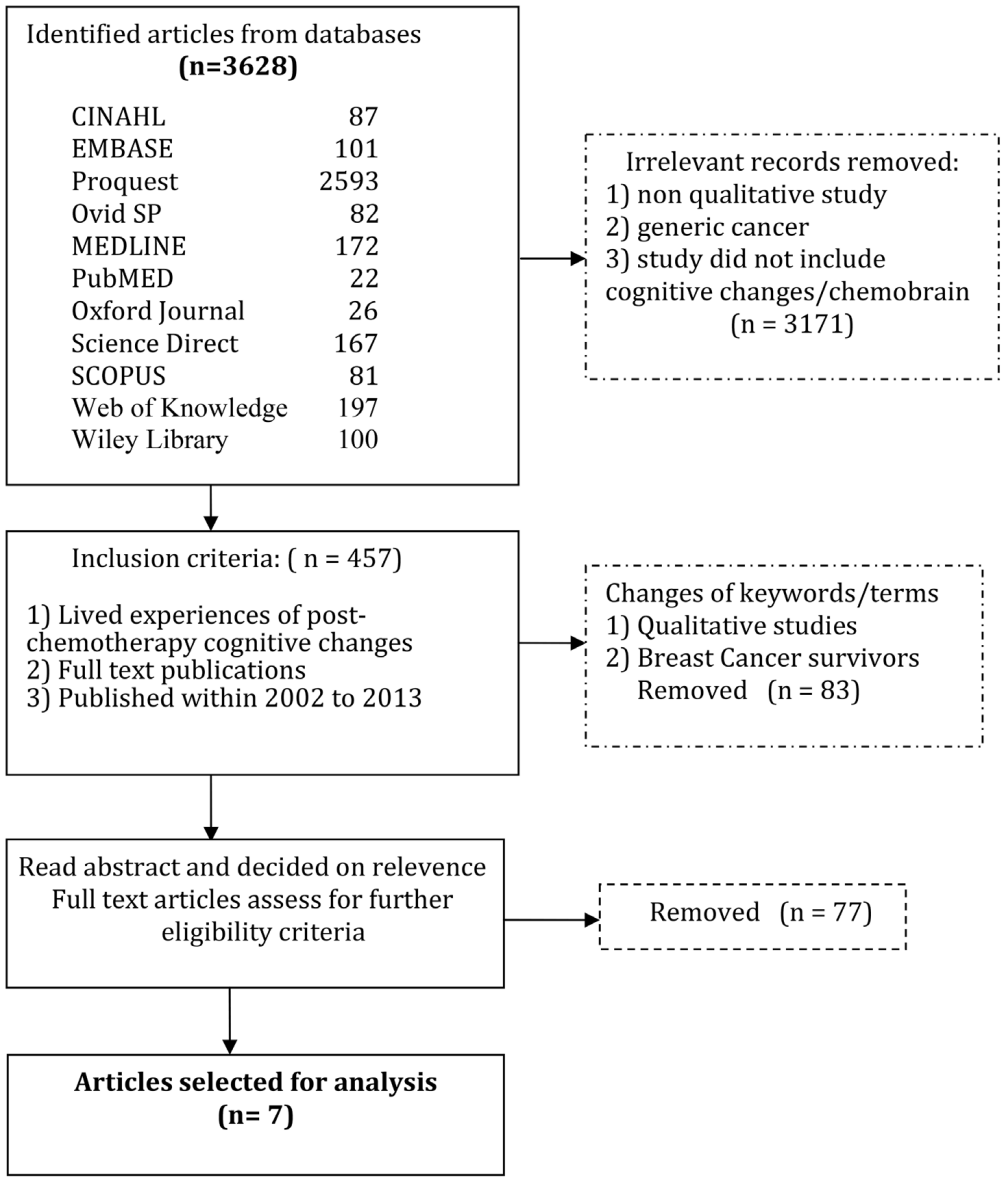
This is the **Figure 1** Search strategy.

The second step of the Noblit and Hare process [23] involved deciding what studies were relevant to our research question and aims. In particular, the research team were interested to understand women’s lived experience of chemobrain. The process of the search strategy is detailed in Figure 1.

### Quality Appraisal

Quality appraisal instruments are available to apply to qualitative research reviews in a consistent manner, to ensure credibility or trustworthiness of the findings [26]. We adopted the ‘Critical Appraisal Skills Program’, or CASP [27] format to appraise each article in order to apply a valid, reliable and objective method prior to synthesis. The CASP checklist is a 10-item tool to appraise qualitative papers without using a numerical score. This is to remind researchers of the importance of every criterion in a qualitative paper in order to reduce bias. Bias in qualitative research is usually related to the influence of researchers and a lack of transparency in data collection and analysis, whereas in positivist quantitative studies, bias is related to representativeness and generalizability. Therefore, qualitative studies are usually evaluated using agreed criteria about data collection and analysis processes that need to be fulfilled in order to contribute to a review of findings. The first two questions act as the core criteria in selecting the qualitative reports [28]. Nevertheless, the process itself is useful as it may contribute to the richness and the focus of discussion [23]. Appraisal of the selected studies contributed to the synthesis of the findings of the review, as any critique of the studies could be identified [29] and incorporated into the review. Table 1 outlines the CASP criteria used to evaluate quality. The first two questions are screening questions. If the answer to both is “yes”, it is worth proceeding with the remaining questions.

**Table 1:** Critical Appraisal Skill Program (CASP Quality appraisal criteria).

**1. Was there a clear statement of the aims of the research?** **2.** **Is a qualitative methodology appropriate?**3. Was the research design appropriate to address the aims of the research?4. Was the recruitment strategy appropriate to the aims of the research?5. Were the data collected in a way that addressed the research issue?6. Has the relationship between researcher and survivors been adequately considered?7. Have ethical issues been taken into consideration?8. Was the data analysis sufficiently rigorous?9. Is there a clear statement of findings?10. How valuable is the research?

The first two questions are screening questions. If the answer to both is “yes”, it is worth proceeding with the remaining questions. Record a “yes”, “no” or “cannot tell” to most of the questions.

### Interpretation with an analytic lens

This study used a seven-step process of meta-ethnography as outlined by Noblit and Hare [25]. Two of the authors (MHS & SYL) independently reviewed the identified studies from the search and appraised the selected papers and undertook the seven steps [22] to extract information and synthesize the findings. Next, discussions took place between the reviewers and other authors where discrepancies arose from collecting and categorising data of each selected study to form the primary dataset. Table 2 outlines the idea of the first process, or gathering of all concepts across the studies, based on subjects’ experiences, as reported by each of the study authors and suggested by Schultz [30]. During this early stage, interpretation was avoided to maintain the original outcomes of each article.

**Table 2:** Critical Appraisal Skill Program (CASP)’s Quality appraisal for selected papers.

CASP Criteria from Table 1	[1]	[2]	[3]	[4]	[5]	[6]	[7]	[8]	[9]	[10]
Mulrooney Tamsin 2007 [32]	Y	Y	Y	Y	Y	Y	Y	Y	Y	Y
Von Ah et al. 2013 [33]	Y	Y	Y	Y	Y	N	Y	Y	Y	Y
Cheung et al. 2012 [34]	Y	Y	Y	Y	Y	Y	Y	Y	Y	Y
Munir et al. [35]	Y	Y	Y	Y	Y	Y	Y	Y	Y	Y
Myers 2012 [36]	Y	Y	Y	Y	Y	Y	Y	Y	Y	Y
Boykoff et al. 2009 [37]	Y	Y	Y	Y	Y	N	Y	Y	Y	Y
Thielen 2008 [38]	Y	Y	Y	Y	Y	N	Y	Y	Y	Y

Index N = No, Y = Yes, C = Cannot tell.

During steps three to five (Noblit and Hare 1988) the research team identified key concepts through reciprocal translation (similar findings) and refutational translation (conflicting findings) and the interpretation of findings were taken together. These processes of explicitly relating the papers occurs on three levels [30]. We systematically followed Schultz’s process to interpret and explain the results via analysis of key methaphors, and were careful not to reduce the qualitative accents but to retain the sense of the account of the women as reported in the papers. Schultz’s concepts of first-order refer to the daily perceptions of the common people; the second-order construct refers to the constructs of findings from the scientific view in the papers, and the third order constructs are final synthesis derived from the key concepts and second order contructs based on the interpetations of the review authors [24].

### Theoretical framework for the review: The Illness Representation Theory

To guide the synthesis for this review, the Model of Illness Representation by Laventhal et al. [31] was adopted. This allowed researchers to capture individual perceptions of illness related to cognitive impairments experienced. This model consists of seven components of illness representation: 1) *Identity*, or the name or label of a threat; 2) *Timeline*, or the belief about the time trajectory for the illness; 3) *Consequences*, or the perceived consequence of a threat from the illness; 4) *Cause*, or the perceived causal mechanism of any threats; 5) *Control/Cure*, or whether something can be done to control the threat; 6) *Illness Coherence*, or whether a person thinks about the threat in a coherent way, and 7) *Emotional representation*, or the emotions associated with the illness experience. This framework is particularly relevant to the review topic and enabled the researchers to interpret and capture the reasoning and self management issues for women experiencing chemobrain.

### Researcher positionality

Inevitably the interpretations of the study data from the review will be influenced in some way by the interests and experiences of the researchers, as researchers were the instruments of both the data collection and the data analysis for the review. Therefore, it was important that the researchers reflected on their own biases in order to overcome them in the data analysis process. MH contributed the theoretical perspectives for the review, SYL is experienced in self management of women following breast cancer, JV contributed medical and clinical encounters with women being treated for breast cancer and LM brought the perspective of an expert-patient with personal experience of breast cancer.

## Results

### Qualitative studies identified for review

A total of 3628 papers were identified electronically following the search using the keywords “chemobrain” and “chemotherapy” and “mild cognitive impairment”. These papers were further scanned for their relevance and this process reduced the number to 457 articles. Next, the criteria for inclusion were applied to the abstracts and this resulted in 83 articles. Further examination of the full versions of these papers, according to the boundaries set by the focused research aim for this study resulted in seven papers that met the inclusion criteria for the review. Some of the qualitative papers did not include the study design in the titles of the publications, so it was necessary for the researchers to read and screen the methodology sections to ensure that a qualitative design was used. The full text for each of these seven papers was obtained and each study was appraised. Figure 1 outlines the search results for the review.

### Results of the quality appraisal process

Based on the CASP checklist (Table 1) as outlined in the methodology section, five of the seven papers were considered to have met all the quality criteria as judged by the reviewers. Table 2 below showed the result of the critical appraisal skill program (CASP)’s quality appraisal for the seven selected papers. The researchers record a “yes”, “no” or “cannot tell” to each questions and then discuss to reach a consensus. Two studies did not meet one quality criterion, which concerned the adequacy of consideration of the relationship between researchers and survivors. These two studies were still included in the review since the unmet criterion was judged to be a minor issue and did not adversely affect the relevance of the studies to the objectives of this review.

### Results of the qualitative review of data extracted

Table 3 outlines the summary of each of the selected papers for review and identifies the themes from each individual study. Based on the synthesis-of-order [25], [26], [29], [30] process, this table summarises the first order constructs that reflect the understandings of the participants from each study and are usually summarised in the results section of each article. Four themes emerged from the analysis of the seven selected papers-: i) The chemobrain struggles, ii) The substantial impact from chemobrain, iii) The struggle to adjust and self manage and iv) The ‘thankful yet fearful’ attitudinal representation. Four second order intepretations were identified, on the basis of which four third order intrepretations (based on key concepts and second-order interpretations) were constructed. These were all linked together in a line of argument that accounts for survivors struggles to self-manage the chemobrain impairment that impacted their activities of daily living.

**Table 3:** Characteristics of the selected studies.

	Aim	Sample & method	Findings	Conclusion	Limitation
Von Ah et al. (2013) USA. [31]	To obtain a better understanding of breast cancer survivors’ experiences of perceived cognitive impairment, its trajectory, and its impact on relationship, daily functioning, work and overall life satisfaction after breast cancer diagnosis and treatment.	**n = 22** breast cancer survivors who reported cognitive impairment at least 1 year postchemotherapy treatment. **Interview** and content analysis approach.	Expressed concern in 6 major domains of cognition: short term memory, long term memory, speed of processing, attention and concentration, language and executive functioning. Chemobrain is frustrating, affects self-confidence and social relationships. Difficulties in work and adapt using compensatory strategies. Validation of perceived cognitive impairment is important for adjustment.	Perceived cognitive deficits have broad implications for wellbeing. Study provides direction for theory development, measurement selection and additional targets. Greater understanding leads to development of effective treatment of these symptoms.	Limited by sample characteristics (geographic area and homogenous). Self report might be influenced by previous participation in cognitive behavioral trial.
Myers, (2012) USA. [34]	To provide an in depth description of the experience of chemotherapy-related cognitive impairment for women with breast cancer and identify related information that women would find useful prior to chemotherapy and cognitive changes	**n = 18** breast cancer survivors who reported cognitive changes within 6–12months postchemotherapy. **Focus group discussion**, semi structured interview and content analysis approach.	Survivors describe difficulty of cogntive changes and the impact in daily living. Survivors shares their coping skills strategies. Survivors want to get information prior to intiating chemotherapy and psychosocial education.	It provides a framework for better understanding regarding the changes that can be used as a guide for patient and family education and generates questions for additional research.	Coding was performed by single investigator and as such may be biased. Interpretation by only one individual poses bias.
Cheung et al. (2012) Singapore. [32]	To gather descriptions from multiethnic breast cancer survivors on their experiences and impact of chemotherapy- associated cognitive changes on daily lives and the coping strategies.	**n = 43** breast cancer patient receiving chemotherapy- **Focus group discussion** and thematic analysis.	Survivors were unfamiliar with the term’chemobrain’ and viewed it as a result of physical and psychosocial adverse effects. Encoutered memory loss, difficulty in decision making, speech problems. Married women claimed frustrations that limited their role as homemaker. Self-identification of coping strategies.	This phenomenon is unfamiliar to most Asians yet it impacted their daily lives. Results suggested that a culturally relevant approach should be adopted to evaluate and manage cognitive changes in these patients.	Selection bias due to nonrandomized sample recruitment and response rate was low. No baseline assessment was conducted. Heterogenous group. Priming effects and preexisting knowledge of chemobrain.
Munir et al. (2009) UK. [33]	To investigate women’s awareness of chemotherapy-induced cognitive changes, their perception of cognitive limitations in carrying out daily tasks and subsequent return to work decision and perception of work ability.	n** = 13 breast cancer** survivors who completed chemotherapy between 12 months to 10 years ago who have returned to work. **Semi structured interview** with two focus groups. Using template analysis.	Survivors noticed decline lasting about a year or longer in concentration, confusion and lack of clear thinking. Chemobrain negatively affects self confidence in cognitive ability and return to work, but support from collegues and employers increased confident in cognitive skills. Impact related to work ability: poor memory, concentration, difficulties in thinking quickly, organising information and decision making. Insufficient information regarding cognitive side effects from oncology team or support groups.	Chemotherapy-induced cognitive impairment affected returning to work and subsequent work ability. Return to work and ability to manage work were influenced by three interrelated factors: 1) actual cognitive ability following chemotherapy, (2) awareness of cognitive failures by the women and their families, & 3) subsequent impact on their confidence in carrying out daily tasks including work tasks.	This study does not explore issues in sufficient depth.
Boykoff et al. (2009) USA. [35]	To document in-depth the effects that cognitive impairment has on women’s personal and professional lives.	**n = 74 white and African American breast cancer** survivors who experienced side effects at least 1 year beyond completion. Focus group/in-depth interviews and content analysis approach	Cognitive impairment can be problematic for survivors. Survivors reported it diminished quality of life and daily functioning. Survivors suggested a range of coping strategies to manage social and profesional lives.	Chemobrain impacts survivors’ economically, emotionally and interpersonally. More research needed on psychosocials aspect of post treatment symptoms to inform the efforts of medical and mental health communities.	This study was non randomised and participants self nominated for the study.
Thielen (2008) USA. [36]	To explore the lived experiences of the neurological changes women describe while undergoing chemotherapy for breast cancer	n** = 13 breast cancer** patients undergoing or completed adjuvant chemotherapy within 12 months. I**nterviews**. A Descriptive phenomenological method guided analysis	Validated the existence of chemobrain phenomenon Women described it affects daily living. These findings may be useful for designing questionaires, educational products and interventional strategies.	A decrease in cognitive function is multifactorial in origin. The womens’ feelings, meaning and perceptions contribute to the fundamental of the lived phenomenon.	Small sample size: Participants were not of mixed ethnicity: sample were from caucasian women. Inexperienced researcher.
Mulrooney Tamsin (2007) USA. [30]	To describe lived experiences of self reported cognitive impairment in a sample of women who were treated with chemotherapy for breast cancer.	n = 10 women with breast cancer – treated with chemotherapy within last 15–52 months. I**nterviews**. A descriptive and interpretative Gadamerian phenomenological theory	Survivors described problems with memory, learning, concentration, language and multitasking. Incidents of chemobrain could occur at anytime and affected the ability to perform usual activities at home and work. Relationship changed among friends and family Chemobrain caused by necessary treatment of breast cancer. Survival was paramount.	The experiences of chemobrain can impact all aspects of life including work. Despite the belief of chemotherapy as a cause, other factors should be acknowledged.	Small numbers, homogenous participants with similar demographic background, educational levels.

1st order construct - Constructs that reflect participants’ understandings, as reported in the included studies and usually found in the results section of an article.

Table 4 outlines the meta-ethnography process that utilises the Schultz [30] notions of first, second, and third order constructs as the follow-up analysis.

**Table 4:** Synthesis of concepts, with second and third order interpretations.

Concepts	Second order interpretations	Third order interpretations
**Experiences of cognitive changes:** Trajectory of cognitive changes, types of cognitive changes, cognitive domains affected, experiences of cognitive changes, awareness of cognitive changes. **Call for help and support:** Healthcare providers to inform of possible cognitive changes, respond to medical community, how to teach me, Looking for answers in all the wrong places, underwhelming information for an overwhelming experience	(a) Patients want validation that it is real and to be prepared for cognitive changes; want health staff to be proactive in addressing the issue; a strategy viewed as able to reduce tension and frustration of family members also	(b) The chemobrain struggle
**Impact of chemobrain:** Self and social relationship – how I changed, daily functioning, working life, psychosocial, financial, overall life satisfaction, change in all aspects of functioning	(c) Significant impact of chemobrain phenomena on self, family, social circle, daily living and work performances.	(d) The substantial impact of chemobrain across life domains
**Coping:** Trying my best to fit in, coping strategies, adjusting to fit in, prior needs of information on cognitive side effects	(e) Ways of coping derived by survivors with multiple strategies to help themselves to overcome the phenomena.	(f) Struggling to self manage (without support from health professionals)
**Reflect on survivorship:** Thankfulness - I am still alive, Apprehension - what the future holds.	(g) Reflection on survivorship to attain normality and regain function	(h) Thankful for life, yet fearful of the future

2nd order construct interpretations of participants' understandings made by authors of these studies (and usually found in the discussion and conclusion section of an article). 3rd order construct the synthesis of both first and second order constructs into a new model or theory about a phenomenon.

#### i) The chemobrain struggle

Chemobrain or chemotherapy-associated cognitive impairment was reported consistently as a real experience. The signs and symptoms were noticed by women with breast cancer, who described them in various ways. The term *chemobrain* offers a quick reference to a concept that may easily encapsulate a range of experiences and enable women to attribute their experiences to a concrete term. However, chemobrain was experienced as a *struggle* as the manifestation of the signs and symptoms triggered the survivors to continually question its existence, and to question if their experience was ‘real or not’. There was a need to seek confirmation via various means, yet the situation remained unresolved as there was no clear answer to their question, leading to a persistent struggle within themselves and with significant others.

Most women felt that chemobrain was an outcome of cancer and its treatment, and perceived themselves to be “chemobrain victims” [32]. Studies used indirect probes to gain descriptions of the experience of chemobrain. and these validated the cognitive changes by providing descriptions such as not being ‘as-sharp or quick’ as before, or feeling ‘foggy’ or ‘spacey’ after treatment [33]. In a study involving Asian women, participants were only able to identify the term chemobrain or describe the symptoms when they were being indirectly probed about their experiences [34]. Survivors noticed cognitive changes during and after chemotherapy treatment [35]. Most of the breast cancer survivors reported changes in the cognitive domains of short and long term memory, processing speed, attention, concentration, language, verbal memory and executive functioning [32], [33], [35], [36].

Although other confounding factors (e.g. cancer related fatigue, mood changes, lack of mental and physical activity, the cancer condition, ageing, hormonal therapy, lack of social support and menopausal status [33], [34], have been found to induce or worsen sustained cognitive issues, many survivors disagreed with this. They justified that they had problems with attention and concentration, although they agreed that they may be more easily distracted when they were tired [33], [34]. Multi-tasking in particular seemed to cause concern and often resulted in feelings of anxiety and frustration [32], [35]. Survivors often described chemobrain as “frustrating”, “upsetting” and some were frightened by problems in processing new information. This phenomenon was perceived to affect their emotions as they struggled to understand the changes occurring.

### Timing of onset of Chemobrain

The onset of chemobrain has been reported at different times across the illness trajectory. Survivors had difficulty giving an exact time of onset of the chemobrain symptoms; however they seemed to lessen over time but not fully resolve [32], [36]. Some survivors reported they experienced changes in cognitive functioning after diagnosis, during chemotherapy or after one to two months of treatment [33], [34], [36], with most reporting that they continued to experience it after the completion of chemotherapy [33], [34]. A rationale offered was that during chemotherapy, many other acute physical symptoms (such as nausea, vomiting, fatigue) could not be ignored, and so survivors did not focus on the subtler cognitive symptoms. Women tend to be overwhelmed by having to suddenly adjust to the reality of a cancer diagnosis, starting chemotherapy and having a potentially life-threatening illness, so that they initially disregarded changes in memory or attention [33], [37]. The present information suggests that cognitive changes can remain a long-term problem for breast cancer survivors.

Dealing with the symptoms or side effects of cancer treatment is a challenge that requires support from others. Family and friends were regarded as good social support systems who encouraged survivors to engage in activities and return to their role in the community. Healthcare providers were also seen as a potential source of support since they work closely with survivors. It has been reported that many healthcare providers are more nonchalant about the phenomena of chemobrain. Survivors reported that their healthcare providers did not discuss any issues relating to chemobrain [33], [36], [37] and some were insensitive when survivors made complaints about their experiences of cognitive deficits. It seemed that the survivors were dissatisfied as they wanted professional validation of their experience of chemobrain symptoms but they were largely ignored.

Healthcare providers often assumed that reported cognitive changes were due to other variables such as, stress or the natural ageing process, and were quick to label chemobrain as a misnomer [37], [38]. Conversely, some women reported that they did receive general information about the possibility of cognitive side-effects from chemotherapy from the oncologists or oncology nurses [38], although conversations were usually initiated by the survivors as they sought explanations [32]. Women felt that early warnings and validation of these changes from healthcare providers with patient-information could help them cope proactively with these changes [33], [36], [38]. Support groups may help survivors to identify or recognise cognitive changes and to be better prepared [36].

#### ii) A substantial impact of chemobrain across life domains

Women described the impact of chemobrain on themselves, their social relationships, working life and daily living [33]. Survivors reported that family and friends ranged from being supportive to being unconcerned about their chemobrain experiences. Some women reported that the cognitive changes had affected their psychological well-being and they lost selfconfidence and self-esteem in the company of family members and friends. Survivors were often confused by their cognitive changes and then felt misunderstood or embarrassed. Survivors who were homemakers described memory difficulties adversely affecting their roles in the family, and that some family members had a lack of awareness about these changes. Some suggested their difficulties maintaining their homemaker roles were related to their own expectations about what they should be able to achieve [33], [34], [37], [38]. Some families provided considerable support as they understood the issues and were aware of the cognitive changes and this was related to positive cultural values. For instance, Cheung et al. [34] reported that the Asian value for living in communities and having a kindred spirit meant that members of the community became a source of support.

Cognitive changes often impacted on working life and school related activities. Survivors indicated that cognitive impairment affected their confidence in returning to work because returning to work would highlight their problems further and be too challenging [35]. Survivors who had returned to work reported that they were struggling to perform and complete tasks. Their job performance had decreased due to an inability to maintain attention or focus at work, to maintain their thoughts during conversations and inability to comprehend a text without reading it more than once. As a result, work tasks required more time and they were less productive. For some, this difficulty contributed to loss of employment and difficulties finding work [33], [36], [37]. Professional women in jobs that required a high level of cognitive functioning were more negatively affected by chemobrain [32]. They reported that they needed more effort to perform tasks than previously, and that as their jobs required several skill sets that incorporated multiple cognitive domains, this created additional challenges. Survivors found they required more attention to complete work tasks to a sufficient standard [32], [33], [38]. Munir et al. [35] found that survivors sometimes hid cognitive difficulties from their employer. However the findings suggest that good support from employers and colleagues, helped survivors to regain confidence in returning to work.

#### iii) Struggling to adjust and to self manage

While struggling to overcome cognitive changes, often without real support or acknowledgement from health professionals, most survivors developed their own strategies to overcome the effects of chemobrain, to prevent further complications and to help them cope with daily living and work functioning These are listed in Table 5. Two studies out of the seven reviewed [33], [35] did not discuss any coping strategies.

**Table 5:** Coping strategies adopted by survivors.

COPING STRATEGIES	32	34	36	37	38
**Pharmacological**					
Nutritional products		**X**			
Complementary and alternative medicine		**X**			
**Non Pharmacological**					
Healthy lifestyle practices		**X**	**X**		
Physical activities		**X**	**X**		
Mental activities	**x**	**x**	**X**		
**Practical reminders**					
**Written**	**X**	**X**	**X**	**X**	**x**
**Use of technology**	**x**	**x**			

The majority of the survivors used non pharmacological strategies such as mental activities, psychosocial management and practical reminders, while some survivors trained themselves in memory strategies to remember things more easily. In the Asian study by Cheung et al. [34] the use of complementary or alternative medicine was more popular than pharmacological intervention as participants perceived these remedies would enhance energy and improve blood circulation to the brain. Coping strategies for some survivors meant avoiding situations that required them to remember names and engage in social conversation. Many survivors decided not to focus on their disabilities and adapted their enviroment to cope with chemobrain by telling people about their cognitive changes [37]. Other survivors relied on their family members and co-workers to remind them about important things [36], [37].

#### iv) Thankful for life, yet fearful of the future

In spite of the challenges of daily living activities, the psychosocial impact, and the lack of support from many health providers during the chemobrain experience, these factors did not cause women with breast cancer to withdraw from chemotherapy treatment. Survivors appreciated receiving chemotherapy as it reduced their risk of mortality. Most were grateful that they had survived and some took their diagnosis as a turning point in their life. They worked towards personal goals for self-satisfaction and some women placed a higher priority on developing relationships with family and friends and contributing to wider society [33], [34].

*“Life isn’t guaranteed, and I try not to be real pessimistic, but it is sort of a wakeup call. You are not guaranteed that tomorrow will come”* [32], p. 122.

However, Thielen [38] reported that survivors were still living in doubt about their cancer prognosis and the likely duration of their chemobrain symptoms. They were apprehensive about the situation as they had received little information about chemobrain. This created additional stress for survivors who were attempting to self-manage what they saw as obvious impairments, which were not acknowledged by their health providers. This also led them to adopt the belief that they should be grateful (to be alive), and ‘downplay’ cognitive impairment as a lesser issue to self manage their return to their normal state.

### Application of the experience of chemobrain to the Leventhal [31] framework

The line-of argument synthesis [24] using the Laventhal model enabled us to frame the significance of the burden of cognitive impairment in cancer survivors. In Table 6 we attempted to present each dimension of Illness Representation Theory [31] in the context of the experiences of chemobrain from the reviewed studies. The cognitive and emotional processes experienced by women were captured and may influence their mental image of potential threats attributable to chemobrain. The interpretation that chemobrain is a relatively small issue (in comparison with death for example), may underestimate the threat of the debilitating effect chemobrain can have on everyday functioning and quality of life. The burden of cognitive impairment in cancer survivors appeared grossly underestimated. This warrants it to be addressed promptly as studies have shown that illness perceptions have associations with a number of negative outcomes in the experience of chronic illness including self-management behaviours and quality of life [39], and this can be anticipated within the breast cancer population.

**Table 6:** Interpretation based on Illness Representation Theory – the struggle of Chemobrain.

Dimension	Potential Manifestations (per illness representation theory)
1. Identity	Matching or nonmatching of cognitive symptoms to the chemobrain experience (e.g., matching symptoms like feeling foggy, not as sharp, not as quick refering to the chemobrain syndrome; or rationalizing it as ‘deficits are unimportant compared to getting through treatment, surviving from cancer)
2. Timeline	Beliefs about the expected onset/duration of it (e.g., acute vs chronic or cyclical). Increasing reports of survivors belief that chemobrain starts after they resume work, but most are unsure of exact timing. Some belief it is transient but others fear its persistent impact. There will be some degree of struggle- transient to persistent
3. Consequences	The perceived and anticipated impact of chemobrain (e.g., reversible vs static vs progressive or permanent). Subjects highlighted that chemobrain affected their daily functioning, economic status, and their social relationships. Some believed these impacts may get better whilst others feared that the cognitive deficits may be permanent loses. The underlying finding is that they will have to struggle with it.
4. Causes	They perceived the contextual factors or antecedent causes (e.g., aging, stress of having cancer and treatment, rather than from treatment since there is no evidence on the mechanism and since their health providers did not validate chemobrain) leading to a constant struggle.
5. Controllability	An expectation that chemobrain symptoms can be somewhat controlled via coping strategies, but may not be cured and may even be permanent damage. Survivors were struggling to adjust. A belief that they should just self-manage since “it does not seems to be a significant according to the health providers’.
6. Illness coherence	The subjects’ perceived understanding of the chemobrain phenomena – (ie vague, subtle, foggy, spacey) but the health team did not validate it, suggesting a period of uncertain struggle.
7. Emotional representa-tions	Panic and frustrated in response to chemobrain experienceSense of dissatisfaction and anger that they were not forewarnedNeutral or matter-of-fact emotional state (for some)Again mansifesting a constant struggle within themselves and with significant others

Based on the Leventhal’s Common Sense Model of Illness Representation [31], [40].

## Discussion

### Implications of the experience of chemobrain

The meta-ethnography method facilitated the synthesis of seven qualitative research studies, and the creation of a preliminary notion to highlight the perceptions women with breast cancer have about their experience of chemobrain. The application of the Illness Representation Theory to the findings allowed further interpretation of the meanings attributed to the chemobrain experience. Although our analytical approach was inductive in nature, it was helpful to consider a theoretical framework that addressed meaning such as the construct of illness representation. The Illness Representation Theory informed the analysis about the ‘vague but real’ cognitive symptoms experienced by breast cancer survivors, portrayed as broader health problems involving identity, timeline, consequences, cause, control, illness coherence, and emotional representations [31]. The idea of illness perceptions is derived from self-regulation theory [31], [40]. This theory proposes that individuals form common-sense beliefs about their symptoms to cope with any potential health threats. Our review suggests that the breast cancer survivors were actively trying to find some meaning to their chemobrain symptoms which became more confronting when they resumed their daily activities.

Illness Representation Theory dictates that illness representations are mediated by influencing factors that have not been adequately explained by the biomedical model of illness. A strong thread of ‘struggling’ with the ‘why’, ‘how’ and ‘when’ of chemobrain is manisfested cognitively and in survivors’ self management behaviour. There is thus, a real gap in survivorship care, as chemobrain becomes a consistently reported phenomena associated with the rise in breast cancer survival rates. Coupled with the unusual terminology of *‘chemobrain’*, survivors have expressed that their health providers are negating its presence, and are therefore not validating its existence. Common sense reasoning is then left to fill the gap, so that cognitive issues are attributed to stress or aging or other potential contextual factors. This leads the survivors to arrive at a state of problem-solving where they struggle to self manage an ‘unknown’ but real issue. The idea that women should just manage as well as they can suggests a constant struggle to cope. The final dimension of illness representation is a cognitive representation of the issue, and this amounts to chemobrain being considered insignificant compared to surviving cancer, despite dissatisfaction with the situation and having to live with the consequences.

### Implications for health services across health settings

Chemobrain or mild cognitive impairment associated with chemotherapy treatment is consistently reported by breast cancer survivors. There is a growing body of research, but the incidence and causes remain uncertain due to inconsistent methods of objective assessment of cognitive changes and subjective reports from survivors [41], [42]. Although many studies suggest that chemobrain is related to cancer treatment such as chemotherapy [43], [44], it is essential to recognise the other confounding factors such as psychological factors and insomnia that could contribute to cognitive changes [45]. In order to enhance the quality of life of survivors, healthcare providers should be reliable sources of information about cognitive changes. A proactive inter-disciplinary team approach comprising the oncology medical staff and allied health professionals is essential to ensure a holistic partnership to provide better care, and to address the participation needs of cancer patients [46].

A consistent evaluation of potential causative factors by health professionals can help to explore the main causes and mechanisms of chemobrain and assist in developing interventions for survivors [46], however few studies have been completed to guide this development. It must be highlighted that some medical clinicians are reluctant to provide information on chemobrain, because of the belief that patients may reject chemotherapy if they believe there is a causal relationship between chemobrain and chemotherapy. Whilst the debate continues, women with breast cancer may face dilemmas about continuing treatment which may lead to worse adverse effects [8] Nevertheless, there are no data to suggest that fears about chemobrain are likely to lead to withdrawal from chemotherapy. In fact, the majority of participants indicated they were willing to undergo chemotherapy despite side effects [33], [34], [36]. However most of the participants stressed the importance of getting early information regarding potential cognitive changes. This needs to be a component of the informed consent process prior to treatment. Our review suggests that most survivors acknowledged their fears but were pragmatic and wanted to be informed early.

### Addressing cognitive impairments

Despite the need for information about chemobrain for survivors, there are other needs to be considered. There is the potential for some psychological consequences of perceiving a threat from chemobrain which may be induced by the provision of information about it [47]. Health care professionals do acknowledge cognitive changes as an issue for the survivors, although there is a lack of scientific data regarding the aetiology [48] Health care professionals, particularly oncologists, tend to emphasise the management of physical side effects [49] or acute side effects which have been reported by patients, as these are more established in the literature.

#### Asian and western perspectives of women with breast cancer

Based on the qualitative papers available in the review, the researchers found emerging differences in approach to their chemobrain experience between the Asian and western subjects. Our review suggested that Asian women are less familiar with the chemobrain phenomena than their western counterparts, however this needs to be interpreted with caution as it was based on only one paper with a small sample size [34]. Asian cultures were emotionally less expressive in emotion [50]. It has been suggested that Asian women are more focused on completing their treatment rather than being concerned with survivorship issues [34], [40]. Nevertheless, the uniqueness of the perceptions of Asian women should be taken into account in any discussion on the issue of chemobrain. These women clearly verified that chemobrain was not a myth, but a real daily experience for them, which became apparent when they resumed daily duties. Many expressed that they were thankful to be alive, and had adopted the belief that chemobrain was just one of the changes that they need to adapt to since survival was the ultimate outcome in overcoming breast cancer. This tolerance of chemobrain symptoms may be best explained by the medicalization of cancer treatment, and this has contributed to the lack of recognition of chemobrain from the medical fraternity.

For both Asian and western cancer survivors, medicalization of the cancer journey is focused on efforts to battle the disease and at the same time is a reminder to survivors that there is a risk of cancer recurrence.

*‘… undermining efforts at self-determination and self-care; and, keeping the patient's life suspended by continual reminders that death is just around the corner, and that all the time and energy left must be devoted to ferreting out and killing the disease’* [40], p.53.

The refusal or reluctance by some medical practitioners to acknowledge the cognitive issues experienced by survivors may lead to a lack of referrals to allied health professionals to address issues like chemobrain. A lack of attention to chemobrain means that the measurement of chemobrain symptoms is neglected. This means that the extent of impairment cannot be defined, and may decrease the impetus to develop effective interventions as outcomes cannot be evaluated. This is a particular issue for the management of return to work for survivors.

### Self-management of chemobrain symptoms

Our review identified a lack of self-awareness about when changes in cognitive functioning became obvious for survivors. It seemed to be most apparent when they struggled to get work done, engaged in their role as a home maker, socialised and tried to cope with being less productive at work. Chemobrain led to specific challenges in handling daily tasks. According to Vearncombe et al. [46] the greatest decline was experienced in verbal learning and memory for breast cancer survivors, with problems in concentration and memory functions, contributing to a decline in functional performance. Inability to perform functional tasks that required constant effort for survivors had resulted in emotional frustration, mood changes and higher anxiety.

Apart from the personal difficulties experienced, our review found that the impact of chemobrain extended to affecting survivors’ social relationships. Survivors reported that they withdrew from social situations to avoid feelings of embarrassment about the effects of their chemobrain symptoms, and this caused changes in their social relationships among family members, friends and colleagues. If they had been prepared and had some self-management guidance, these social consequences may have been lessened or prevented.

In terms of self-managing chemobrain, cancer survivors struggled and tried many ways to cope with impairments. Most of the selected studies found that psychosocial interventions and practical reminders were good sources of coping strategies. There appeared to be some cultural differences in coping strategies for chemobrain. Asian women were more likely to use complementary and alternative medicine such as traditional Chinese medicine to improve their cognitive function [34]. Further studies are needed to explore how cultural beliefs and health setting models can influence early health intervention and the coping strategies of survivors. Research exploring how ethnicity or cultural background can affect how someone copes with chemobrain symptoms, and how they go about dealing with the situation, is a viable and timely topic to minimise the gap in this field of cancer survivorship studies.

### Reflectivity of conducting the metaethnography study

We did encounter some challenges in developing a new synthesis of qualtitative research of a meta-ethnography study on ‘chemobrain, in particular in synthesizing methods from different contexts and research traditions. Some issues we encountered were the need to appraise the papers to ensure they fit our research question, and the CASP quality.

### Strengths and limitations of the study

The strength of this review is the direct insights obtained from reports by breast cancer survivors about the cognitive changes they experienced in real life. Synthesizing the data from various qualitative researchers helped identify the scope and depth of the key domains of cognitive impairments experienced across different cohorts, time periods, cultures and health settings using a variety of qualitative study methodologies. Using the meta-ethnography has allowed a body of qualitative research studies to be combined together in a systematic way, where we have attempted to inductively analyse them through extracting concepts, metaphors and themes arising from the seven different studies. Meta ethnography has been proven useful where reflection on such interpretative approaches are needed and it has been used widely in health studies [22], [23], [51], [52]. In short, the strength of this approach lies in the way it was constructed to preserve the meaning of the primary data and the help it gives us to look into a theoretical underpinning to understand the chemobrain phenomenon in a new intepretation of synthesis [53], [54]. However, whilst the method followed a systematic process, it remained fundamentally inductive as it re-interpreted qualitative data presented across several qualitative studies. Therefore, we conclude that these findings can inform the development of recommendations for health care professionals, including clinical practice guidelines on patient education, assessment and interventions for breast cancer survivors to address chemobrain symptoms.

Several limitations have been acknowledged in this review study. We recognised the importance of maximum variation sampling, however the number of studies included and the sample size within each study was small. The inclusion criterion of published sources with full text availability may have omitted the inclusion of other potential sources such as unpublished theses or published book chapters, and this may have reduced the richness of the data. Five out of seven of the studies were from the United States, one was from the United Kingdom and one was from the Asian region. There is clearly a gap in studies from many other geographical locations. Future studies should explore the differences between less developed countries and developed countries, where the healthcare systems are uniquely different. Despite the limitations, meta-ethnography is a useful method for synthesising qualitative research conducted on a specific issue, and for developing models that interpret findings across multiple studies [28].

## Conclusion

Our review found clear verification and consistent reports that breast cancer survivors’ experiences of cognitive impairments were real, with a reported disparity between health professional and survivor’s viewpoints across different healthcare settings and cultures. Persistent chemobrain clearly has a detrimental impact on the economic, emotional and interpersonal status of breast cancer survivors. Subjective self-report were validated by the consistent findings across the studies, and it is likely that the severity of difficulties experienced (manifested as ‘strugglings to self manage’) was underestimated. Women may downplay the effect of these impacts because of the lack of recognition from health providers. The current literature on the lived experience of chemobrain symptoms by women with breast cancer provides (manifesting as a constant struggle on their daily living domains) evidence that chemobrain is real, persistent and has substantial, detrimental impacts on daily living and quality of life.

### Recommendations

On the basis of the key findings discussed above, we recommend the development of an information resource to create awareness of potential cognitive changes associated with breast cancer treatment among patients, family caregivers and, perhaps most importantly, among healthcare providers. A more objective testing and monitoring of neurocognitive function in survivors complaining of cognitive changes is warranted. In addition, studies evaluating cognitive testing should be derived from reliable, functional and ecologically valid assessments which are culturally defined, rather than depending only on pen and paper assessments. Optimisation of some of the self-management strategies used by the some breast cancer survivors can be used to inform the development of educational information for survivors, and for enhancing awareness among healthcare providers. Further quantitative research is required to explore the causal mechanisms associated with chemobrain symptoms. Future research that includes systematic, longitudinal investigations of illness representation and its impact on health behaviours among survivors with cognitive impairment is needed. Greater awareness and culturally-specific therapy is critical to enhance functional return to daily tasks and quality of life during the cancer survivorship phase, especially related to return to work. Our study findings contribute to both theoretical and empirical implications for future research and the development of practice to address cognition in cancer survivorship.
