# Correction: Chemobrain Experienced by Breast Cancer Survivors: A Meta-Ethnography Study Investigating Research and Care Implications

**DOI:** 10.1371/journal.pone.0117740

**Published:** 2015-02-03

**Authors:** The PLOS ONE Staff

There are errors in Table 3 of the published paper. The correct Table 3 can be seen here.

**Table 3:** Characteristics of the selected studies.

	Aim	Sample & method	Findings	Conclusion	Limitation
Von Ah et al.(2013) USA. [33]	To obtain a better understanding of breast cancer survivors’ experiences of perceived cognitive impairment, its trajectory, and its impact on relationship, daily functioning, work and overall life satisfaction after breast cancer diagnosis and treatment.	n = 22 breast cancer survivors who reported cognitive impairment at least 1 year postchemotherapy treatment. Interview and content analysis approach.	Expressed concern in 6 major domains of cognition: short term memory, long term memory, speed of processing, attention and concentration, language and executive functioning. Chemobrain is frustrating, affects self-confidence and social relationships.Difficulties in work and adapt using compensatory strategies. Validation of perceived cognitive impairment is important for adjustment.	Perceived cognitive deficits have broad implications for wellbeing. Study provides direction for theory development, measurement selection and additional targets. Greater understanding leads to development of effective treatment of these symptoms.	Limited by sample characteristics (geographic area and homogenous). Self report might be influenced by previous participation in cognitive behavioral trial.
Myers, (2012) USA. [36]	To provide an in depth description of the experience of chemotherapy-related cognitive impairment for women with breast cancer and identify related information that women would find useful prior to chemotherapy and cognitive changes	n = 18 breast cancer survivors who reported cognitive changes within 6–12months postchemotherapy. Focus group discussion, semi structured interview and content analysis approach.	Survivors describe difficulty of cogntive changes and the impact in daily living.Survivors shares their coping skills strategies. Survivors want to get information prior to intiating chemotherapy and psychosocial education.	It provides a framework for better understanding regarding the changes that can be used as a guide for patient and family education and generates questions for additional research.	Coding was performed by single investigator and as such may be biased. Interpretation by only one individual poses bias.
Cheung et al.(2012) Singapore. [34]	To gather descriptions from multiethnic breast cancer survivors on their experiences and impact of chemotherapy- associated cognitive changes on daily lives and the coping strategies.	n = 43 breast cancer patient receiving chemotherapy- Focus group discussion and thematic analysis.	Survivors were unfamiliar with the term’chemobrain’ and viewed it as a result of physical and psychosocial adverse effects. Encoutered memory loss, difficulty in decision making, speech problems.Married women claimed frustrations that limited their role as homemaker. Self-identification of coping strategies.	This phenomenon is unfamiliar to most Asians yet it impacted their daily lives.Results suggested that a culturally relevant approach should be adopted to evaluate and manage cognitive changes in these patients.	Selection bias due to nonrandomized sample recruitment and response rate was low.No baseline assessment was conducted. Heterogenous group.Priming effects and preexisting knowledge of chemobrain.
Munir et al.(2010) UK. [35]	To investigate women’s awareness of chemotherapy-induced cognitive changes, their perception of cognitive limitations in carrying out daily tasks and subsequent return to work decision and perception of work ability.	n = 13 breast cancer survivors who completed chemotherapy between 12 months to 10 years ago who have returned to work. Semi structured interview with two focus groups. Using template analysis.	Survivors noticed decline lasting about a year or longer in concentration, confusion and lack of clear thinking.Chemobrain negatively affects self confidence in cognitive ability and return to work, but support from collegues and employers increased confident in cognitive skills. Impact related to work ability: poor memory, concentration, difficulties in thinking quickly, organising information and decision making. Insufficient information regarding cognitive side effects from oncology team or support groups.	Chemotherapy-induced cognitive impairment affected returning to work and subsequent work ability. Return to work and ability to manage work were influenced by three interrelated factors: 1) actual cognitive ability following chemotherapy,(2) awareness of cognitive failures by the women and their families, &3) subsequent impact on their confidence in carrying out daily tasks including work tasks.	This study does not explore issues in sufficient depth.
Boykoff et al.(2009) USA.[38]	To document in-depth the effects that cognitive impairment has on women’s personal and professional lives.	n = 74 white and African American breast cancer survivors who experienced side effects at least 1 year beyond completion. Focus group/in-depth interviews and content analysis approach	Cognitive impairment can be problematic for survivors. Survivors reported it diminished quality of life and daily functioning. Survivors suggested a range of coping strategies to manage social and profesional lives.	Chemobrain impacts survivors’ economically, emotionally and interpersonally. More research needed on psychosocials aspect of post treatment symptoms to inform the efforts of medical and mental health communities.	This study was non randomised and participants self nominated for the study.
Thielen(2008) USA.[37]	To explore the lived experiences of the neurological changes women describe while undergoing chemotherapy for breast cancer	n = 13 breast cancer patients undergoing or completed adjuvant chemotherapy within 12 months. Interviews. A Descriptive phenomenological method guided analysis	Validated the existence of chemobrain phenomenon Women described it affects daily living. These findings may be useful for designing questionaires, educational products and interventional strategies.	A decrease in cognitive function is multifactorial in origin. The womens’ feelings, meaning and perceptions contribute to the fundamental of the lived phenomenon.	Small sample size: Participants were not of mixed ethnicity: sample were from caucasian women.Inexperienced researcher.
Mulrooney Tamsin(2007) USA. [32]	To describe lived experiences of self reported cognitive impairment in a sample of women who were treated with chemotherapy for breast cancer.	n = 10 women with breast cancer—treated with chemotherapy within last 15–52 months. Interviews. A descriptive and interpretative Gadamerian phenomenological theory	Survivors described problems with memory, learning, concentration, language and multitasking. Incidents of chemobrain could occur at anytime and affected the ability to perform usual activities at home and work. Relationship changed among friends and family Chemobrain caused by necessary treatment of breast cancer. Survival was paramount.	The experiences of chemobrain can impact all aspects of life including work. Despite the belief of chemotherapy as a cause, other factors should be acknowledged.	Small numbers, homogenous participants with similar demographic background, educational levels.

1st order construct: Constructs that reflect participants' understandings, as reported in the included studies and usually found in the results section of an article.

## References

[1] Selamat MH, Loh SY, Mackenzie L, Vardy J (2014) Chemobrain Experienced by Breast Cancer Survivors: A Meta-Ethnography Study Investigating Research and Care Implications. PLoS ONE 9(9): e108002 doi:10.1371/journal.pone.0108002 25259847 10.1371/journal.pone.0108002 PMC4178068

